# Exploring Heparan Sulfate Proteoglycans as Mediators of Human Mesenchymal Stem Cell Neurogenesis

**DOI:** 10.1007/s10571-024-01463-8

**Published:** 2024-03-28

**Authors:** Sofia I. Petersen, Rachel K. Okolicsanyi, Larisa M. Haupt

**Affiliations:** 1 Stem Cell and Neurogenesis Group, School of Biomedical Sciences, Genomics Research Centre, Centre for Genomics and Personalised Health, Queensland University of Technology (QUT), 60 Musk Ave, Kelvin Grove, QLD 4059 Australia; 2 ARC Training Centre for Cell and Tissue Engineering Technologies, Queensland University of Technology (QUT), Kelvin Grove, Australia; 3 Max Planck Queensland Centre for the Materials Sciences of Extracellular Matrices, Kelvin Grove, Australia

**Keywords:** Heparan sulfate proteoglycan, Alzheimer’s disease, Traumatic brain injury, Human mesenchymal stem cell, Neurogenesis, Amyloid beta

## Abstract

Alzheimer’s disease (AD) and traumatic brain injury (TBI) are major public health issues worldwide, with over 38 million people living with AD and approximately 48 million people (27–69 million) experiencing TBI annually. Neurodegenerative conditions are characterised by the accumulation of neurotoxic amyloid beta (Aβ) and microtubule-associated protein Tau (Tau) with current treatments focused on managing symptoms rather than addressing the underlying cause. Heparan sulfate proteoglycans (HSPGs) are a diverse family of macromolecules that interact with various proteins and ligands and promote neurogenesis, a process where new neural cells are formed from stem cells. The syndecan (SDC) and glypican (GPC) HSPGs have been implicated in AD pathogenesis, acting as drivers of disease, as well as potential therapeutic targets. Human mesenchymal stem cells (hMSCs) provide an attractive therapeutic option for studying and potentially treating neurodegenerative diseases due to their relative ease of isolation and subsequent extensive in vitro expansive potential. Understanding how HSPGs regulate protein aggregation, a key feature of neurodegenerative disorders, is essential to unravelling the underlying disease processes of AD and TBI, as well as any link between these two neurological disorders. Further research may validate HSPG, specifically SDCs or GPCs, use as neurodegenerative disease targets, either via driving hMSC stem cell therapy or direct targeting.

**Graphical Abstract:**

Graphical abstract: Heparan sulfate proteoglycans as regulators of human mesenchymal stem cell neurogenesis. Traumatic brain injury (TBI) and genetic factors increase Alzheimer’s disease (AD) risk (yellow). Potential AD treatment targets (green) include human mesenchymal stem cells (hMSCs). Manipulating pathway and growth factor interactions with heparan sulfate proteoglycans (HSPGs) could regulate hMSC neurogenesis, potentially offering functional neural stem cell transplants as AD treatments

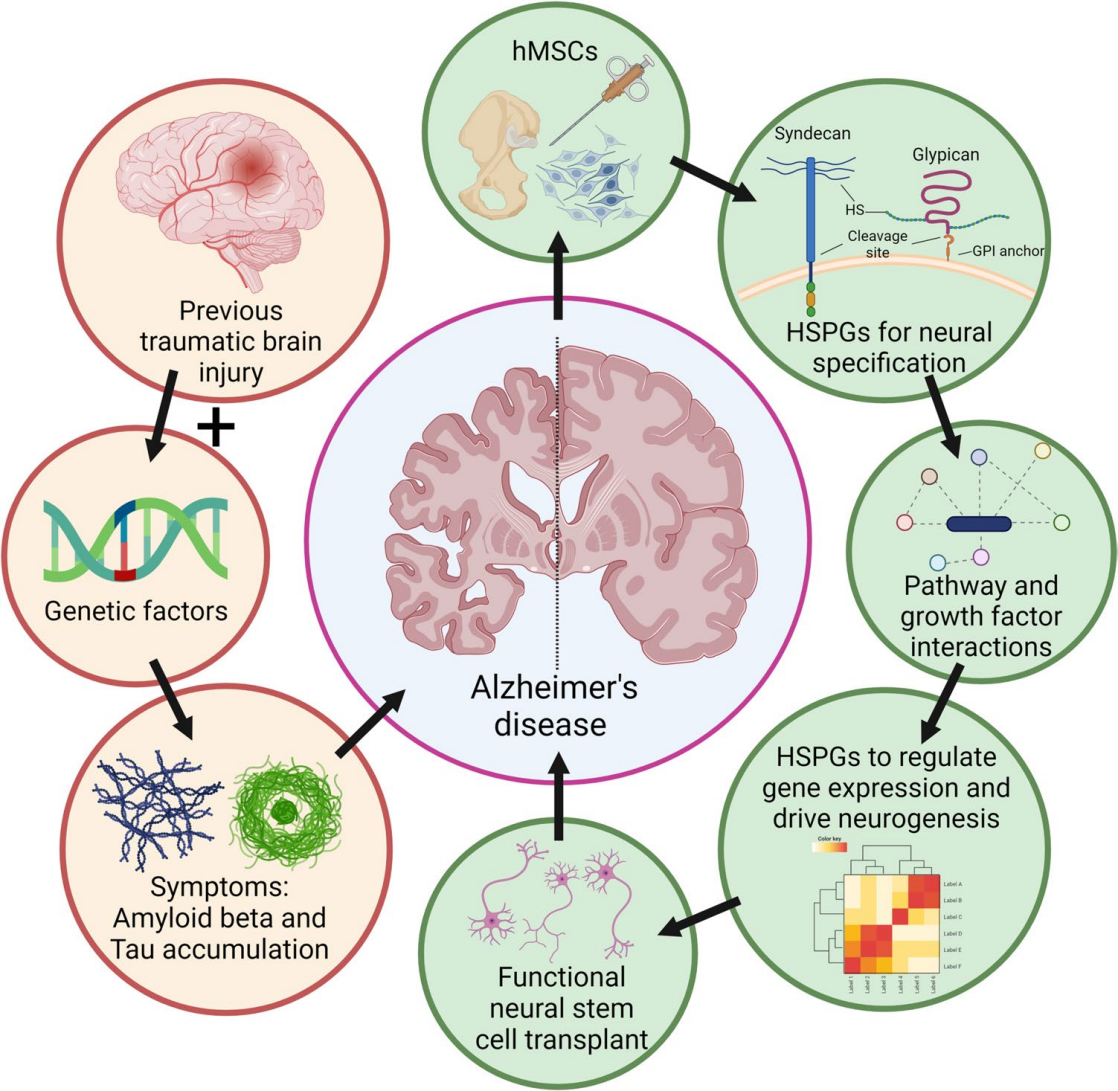

## Alzheimer’s Disease and Traumatic Brain Injury: Neurodegenerative Diseases with No Cure

In 2022, over 38 million people were estimated to be living with Alzheimer’s disease (AD) worldwide (World Health Organization 2023). In addition, between 27 and 69 million people experience a traumatic brain injury (TBI) annually, significantly increasing their risk of developing AD (Dewan et al. 2018). The accumulation of amyloid beta (Aβ) and microtubule-associated protein Tau (Tau) has been linked to both AD and TBI disease models (Golde et al. 2010; Ramos-Cejudo et al. 2018). Current treatment options unsuccessfully target the symptoms of these neurodegenerative diseases and without a treatment the healthcare burden continues to amplify in aging populations worldwide.

AD is characterised by progressive cognitive decline in language, visuospatial and executive function, as well as personality and behavioural changes (Naasan et al. 2021; Weller and Budson 2018). TBI is a heterogeneous condition characterised by similar symptoms, with disease presentation dependant on the injury and its location (McKee and Daneshvar 2015; Najem et al. 2018) The neural and structural damage seen in AD and TBI can affect people of all ages (Dams-O’Connor et al. 2016) with AD and TBI patients demonstrating significant cognitive decline secondary to neuronal death. The application of stem cell transplants to induce repair processes, such as neurogenesis, have the potential to provide improved treatment options for these neurodegenerative disorders (Jullienne et al. 2014; Reiss et al. 2018). Under specific culture conditions and with the use of appropriate growth factors, human mesenchymal stem cells (hMSCs) can be induced to undergo neural differentiation. A deeper understanding underlying the biological processes guiding hMSC differentiation are needed to progress stem cell therapy and their application to neurological repair.

## Heparan Sulfate Proteoglycans

Heparan sulfate proteoglycans (HSPGs) are a diverse family of macromolecules, decorated with multiple glycosaminoglycan (GAG) chains (Okolicsanyi et al. 2014a). These heterogeneous side chains are characterised by specific sulfation patterns, which enable HSPGs to interact with various protein families and ligands, including growth factors, enzymes, lipases and extracellular matrix (ECM) components (Knelson et al. 2014). As most of the current understanding of HSPGS stems from murine models, further investigating neurogenesis from human stem cells and the involvement of HSPGs is essential to advance neural stem cell therapies (Oikari et al. 2014). Two major HSPG families include the four transmembrane syndecans (SDC1-4) and six via Glycosylphosphatidylinositol (GPI)-anchored glypicans (GPC1-6) (Leonova and Galzitskaya 2013; Oikari et al. 2020; Tkachenko et al. 2005). In addition, secreted ECM HSPGs include perlecan, agrin and type XVIII collagen (Sarrazin et al. 2011; Ughy et al. 2019). SDCs have been reported to mediate angiogenesis, tumourigenesis and embryonic growth, while GPCs mediate signalling activity (Okolicsanyi et al. 2015). In particular, syndecan-3 (*SDC3*) and glypican-1 (*GPC1*) have been linked to key features of neurodegenerative disorders—increased intracellular Aβ accumulation and to alteration of amyloid beta precursor protein (*APP*) gene function (Letoha et al. 2019; O’Callaghan et al. 2008; Snow et al. 1994). SDC1 controls hMSC lineage determination in an osteogenic model (Yu et al. 2020) and GPC1 and GPC4 core proteins have been linked to neural lineage commitment in human NSC experiments (Oikari et al. 2016; Yu et al. 2017). While the role of HSPGs in various cellular processes has been extensively investigated, their specific involvement in neural differentiation and neurogenesis of hMSCs remains poorly understood.

## Heparan Sulfate Biosynthesis

Complex temporal biosynthesis within the Golgi apparatus determines the final nature of GAG chains, specifying them as heparan sulfate (HS), dermatan sulfate (DS), keratan sulfate (KS) or chondroitin sulfate (CS) (Christianson and Belting 2014; Couchman et al. 2015). HS consists of *N*-acetyl glucosamine (GlcNAc) and glucuronic acid (GlcA) repeats that are polymerised by the exostosin (EXT) enzymes *EXT1* and *EXT2* (Annaval et al. 2020; Okolicsanyi et al. 2018). C5-epimerase enzyme action improves HS flexibility and protein ligand recognition, enabling protein binding and polymer function (Debarnot et al. 2019; Qin et al. 2015). This process is followed by *N*-sulfation of HS by *N*-Deacetylase/*N*-Sulfotransferase (NDSTs), specifying the chains as HS GAGs (Okolicsanyi et al. 2014a). When the NDSTs are active, a HS chain is formed, however, as with all GAGs, the biosynthesis of HS starts with Exostosin-like 3 (EXTL3), which adds an N-acetylglucosamine residue (GlcNAc) to the glucuronic acid (GlcA) residue onto a tetrasaccharide linker (Okolicsanyi et al. 2014b; McMillan et al. 2023). N-sulfation introduced by NDSTs only occurs as part of HS chain formation and is absent in chondroitin sulfate (CS), dermatan sulfate (DS) and keratan sulfate (KS) chains (Aikawa and Esko 1999; Pikas et al. 2000). The final length and sulfation pattern of GAG chains elicit various functions, including regulation of cellular behaviour, migration and differentiation (Okolicsanyi et al. 2014b). HS side chains stimulate these cellular processes via binding to a wide variety of protein ligands, such as cytokines, enzymes, growth factors and protein structures within the ECM (Sugahara and Kitagawa 2002).

## SDCs and GPCs as Targets for Neurodegenerative Disease Research

The SDCs and GPCs are crucial for mediating cellular functions, including neurogenesis; however, the exact functional mechanisms of these interactions have not yet been fully elucidated. The transmembrane SDCs carry either a HS or both HS and CS chains and consist of an ectodomain at their N-terminal, along with a transmembrane domain and a cytoplasmic domain at their C-terminal (Hudák et al. 2021; Lopes et al. 2006; Xian et al. 2010). Each SDC has distinct expression patterns and functions in their respective tissues. SDC1 expression is mainly found within epithelial and mesenchymal cells, SDC2 in mesenchymal, neuronal and epithelial cells, SDC3 in neuronal and musculoskeletal cells and SDC4 expressed in most tissues (Lambaerts et al. 2009; Tkachenko et al. 2005). SDCs can function as receptors and co-receptors for ECM components and interact with growth factors and other ligands to regulate downstream signalling pathways, regulating cellular behaviours (Xian et al. 2010). Specifically, SDCs have been shown to interact with a variety of growth factor signalling pathways, including the Wnt and fibroblast growth factor (FGF) pathways (Dong et al. 2021; Sakane et al. 2012; Sarrazin et al. 2011).

In contrast, GPCs are covalently linked to the cell surface via GPI anchors and are characterised by their 14 cysteine residues near their *N*-terminus or central domain of the core protein (Awad et al. 2015; De Cat and David 2001; Dong et al. 2021). The cysteine residues may form disulfide bonds intramolecularly, accounting for their globular tertiary structure (De Cat and David 2001; Fico et al. 2011). The GPCs are expressed in various tissue types primarily during development, functioning though interactions with Wnt and FGF signalling pathways (Sarrazin et al. 2011; Wang et al. 2019). GPC1 is expressed in the central nervous system (CNS) in differentiated neurons, while GPC2 is expressed in developing neuronal cells (Lugert et al. 2017; Shi et al. 2021). GPC3 has been found to be ubiquitous during development in cells other than the CNS and GPC4 localised to the kidneys, adrenal tissue and neural precursor cells (Sakane et al. 2012). Low levels of GPC5 expression have been identified in various tissues, including the brain, heart and lungs with GPC5 reported to mediate cell proliferation and migration (Li et al. 2018; Yuan et al. 2016). GPC5 has also been shown to interact with various growth factors and signalling molecules, including Wnt and fibroblast growth factor 2, to modulate their activity (Li et al. 2018; Tkachenko et al. 2005; Yuan et al. 2016). Increased expression of GPC5 has also been correlated with poor prognosis in some cancer types (Li et al. 2018; Yuan et al. 2016). GPC6 has been suggested to be important in neural development and plasticity and to interact with various signalling molecules to modulate their activity (Kamimura and Maeda 2021; Tkachenko et al. 2005). In particular, GPC6 has been shown to bind FGF2 to enhance its signalling, promote neurite outgrowth and neural stem cell differentiation (Filmus et al. 2008; Tkachenko et al. 2005). Investigating SDCs and GPCs in their diverse expression patterns and various tissue types, as well as their interactions with signalling pathways (Wnt, FGF) that regulate neurogenesis will further our understanding of normal and pathological processes. Specifically, the interactions between these pathways and relevant HSPGs, such as “neuronal” *SDC3* and *GPC1*, expressed in differentiated neurons, holds potential for deeper insight into understanding the complex mechanisms regulating human neurogenesis.

## Alzheimer’s Disease Aetiology

The aetiology of AD is complex and remains poorly understood; however, genetic, environmental and lifestyle factors have all been identified to influence AD risk (Breijyeh and Karaman 2020). AD pathogenesis is characterised by the extensive formation of Aβ plaques, neurofibrillary tangles (NFTs) resulting from Tau and hyperphosphorylated Tau (p-Tau) aggregates and vascular amyloid angiopathy (Ramos-Cejudo et al. 2018; Reiss et al. 2018; Solis et al. 2020). These pathogenetic features result in neural cell death and subsequent disease presentation, with stem cell therapy actively pursued as a potential treatment option including via stimulation of neurogenesis. Aβ plaques, p-Tau, total Tau protein and NFTs have long been considered hallmark pathological features of AD development (Horie et al. 2020; Rajmohan and Reddy 2017). In addition, AD disease phenotypes have been linked to neuropil threads, dystrophic neurites, astrogliosis, microglial activation as well as cerebral amyloid angiopathy (Lane et al. 2018). The pathogenesis of AD involves the complex interplay between protein aggregation, cell surface proteins and their receptors. HSPGs, namely SDC and GPC, the focus of this review, have been implicated in various processes driving neurodegeneration as detailed below. Unravelling the processes permissive of AD aetiology, along with the associated signalling pathways and potential HSPG involvement may allow for improved understanding of how these factors may be targeted to slow down or treat neurodegeneration.

## APP Processing and Amyloid Beta Accumulation

**Fig. 1:**
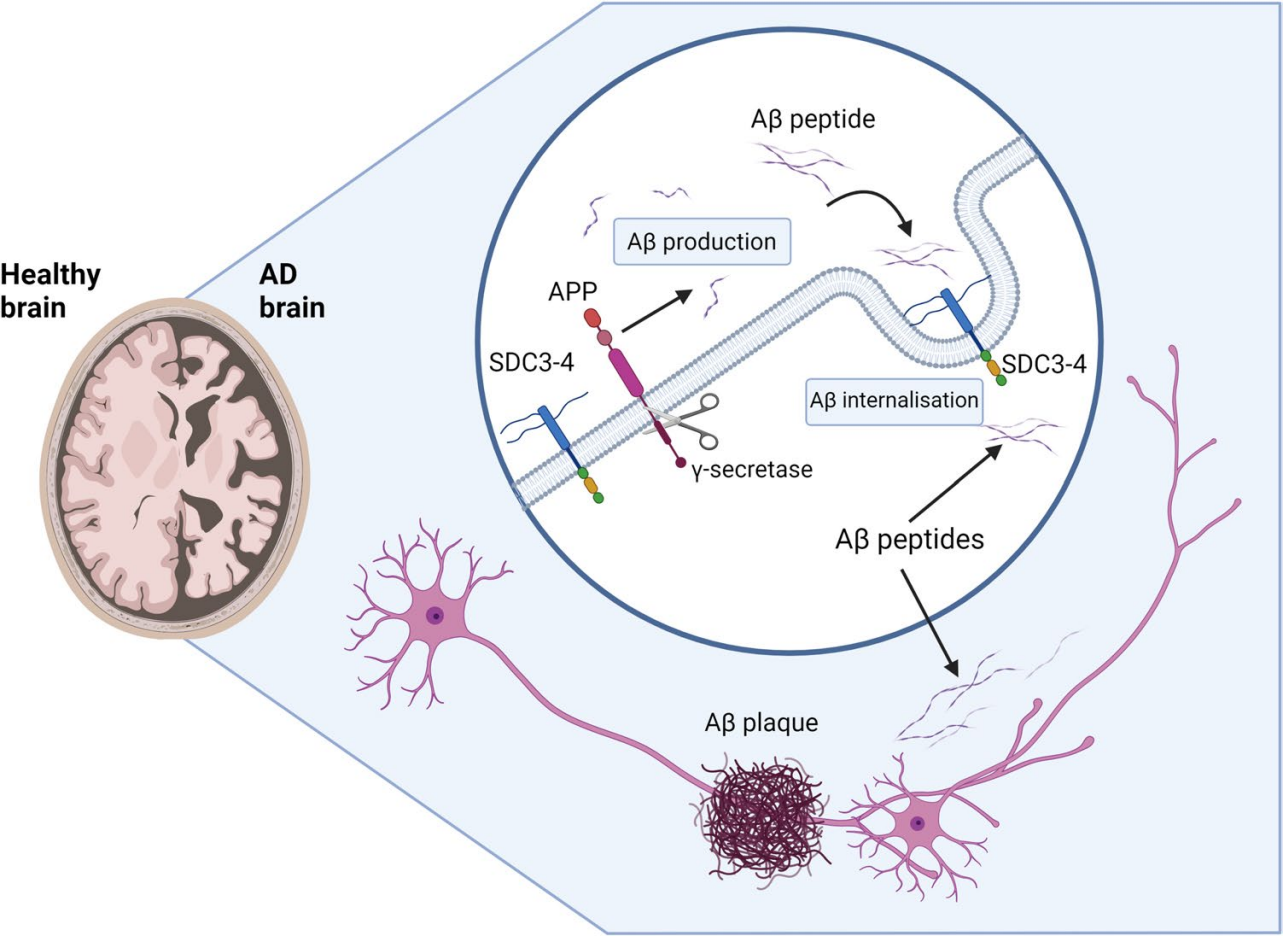
Impaired amyloid precursor protein (APP) cleavage and the resulting formation of amyloid beta (Aβ) oligomers and senile plaques in Alzheimer’s disease (AD). The major pathogenic event in AD is the impaired cleavage of APP by γ-secretase, which leads to the aggregation of Aβ fragments. The impaired cleavage of APP by γ-secretase results in the formation of Aβ peptides, which are internalised and then aggregate to form senile Aβ plaques. The syndecan (SDC) heparan sulfate proteoglycans (HSPGs) are present on the cell membrane and extracellular matrix, and their full mediation of Aβ internalisation remains elusive. SDC3 and SDC4 increase the internalisation of Aβ via attachment to heparan sulfate (HS) chains, with potential contribution to AD pathogenesis. The presence of these neurotoxic aggregates results in the shrinkage of key brain regions, including the cerebral cortex, which is responsible for language, memory, decision making, and personality, and the hippocampus, which is responsible for learning and memory

While AD is widely considered to be polygenically influenced (Williamson et al. 2009), the impaired cleavage of amyloid precursor protein (APP) is currently thought to be the major pathogenic event in the development of the disease (Huse et al. 2002). APP is encoded by the similarly named *APP* gene (O’Brien and Wong 2011), that is ubiquitous in the central nervous system (CNS) and is proteolysed intracellularly (Muller and Zheng 2012; O’Brien and Wong 2011). Impaired cleavage by γ-secretase at the C-terminus of APP produces Aβ fragments, which are internalised and then can accumulate and become neurotoxic (Fig. 1) (Gu and Guo 2013; O’Brien and Wong 2011). Recent evidence suggests that SDCs may contribute to the internalisation of Aβ peptides, however, the mechanism and degree of impact remain unknown (Ozsan McMillan et al. 2023). SDC3 and SDC4 were shown to exhibit enhanced uptake of Aβ1–42 in SH-SY5Y cell models (Letoha et al. 2019).

As such, AD is characterised by the pathological intracellular accumulation of Aβ fragments (Abu Hamdeh et al. 2018). Additionally, extracellular Aβ aggregates can induce hyperphosphorylation of Tau, increasing NFT aggregation (Bettens et al. 2010). The primary constituents of these senile plaques include the insoluble aggregates of the 4-kDa Aβ peptides, Aβ40 and Aβ42, generated by sequential proteolytic processing of APP by β-site APP cleaving enzyme 1 (BACE1) and γ-secretase (O’Brien and Wong 2011; Picone et al. 2020). While Aβ40 is the most abundant Aβ peptide in the cerebrospinal fluid (CSF), the slightly longer and less abundant Aβ42 has been implicated as the key pathogenic species in AD brain (Baldeiras et al. 2018; Gu and Guo 2013). Aβ42 is a major component of amyloid plaques in the AD brain, however Aβ40 has only been detected in trivial amounts (Ferrari and Sorbi 2021). The Aβ42/Aβ40 ratio is considered to be indicative of AD severity and progression, with increased ratios representative of higher neurotoxicity and Tau pathology via induction of Aβ fibril formation (Kumar-Singh et al. 2006; Kuperstein et al. 2010). Several studies have also found that the two isoforms influence aggregation rates and toxicity of each other (Kuperstein et al. 2010; Pauwels et al. 2012; Snyder et al. 1994). Additionally, Aβ42 binding may be preferential in the ECM or cell membrane, leading to increased concentrations (Banerjee et al. 2020; Chen et al. 2017). A recent study found that the structural variability of Aβ42 and Aβ40 fibrils may also affect the phenotypical and clinical differences observed in AD (Banerjee et al. 2020; Chen et al. 2017; Qiang et al. 2017). These differences also may, however, be due to genetic and environmental differences as well as fibril structure (Qiang et al. 2017). Mouse models have demonstrated that HS GAG chains colocalise with Aβ in AD patient brains and interact with Aβ and amyloid fibrils; however, the mechanisms underlying this interaction remains elusive (van Horssen et al. 2003; Zhang et al. 2014). One hypothesis suggests that the HS GAG side chains may allow HSPGs to act as chaperones, protecting Aβ from proteolytic degradation, potentially hindering the clearance of Aβ (Gupta-Bansal et al. 1995; Snow et al. 1994). Furthermore, early studies have identified global expression of HSPGs in human tissue NFTs via immunostaining (Snow et al. 1988; Su et al. 1992), with SDC1-3 and GPC1 core proteins detected in amyloid plaques (van Horssen et al. 2002a, b; Verbeek et al. 1999). Additionally, elevated levels of *SDC3, SDC4, GPC1* and *GPC3* have also been observed in AD patient brain tissues (Liu et al. 2016). Identification of the intricate roles of SDCs and GPCs in amyloid accumulation and AD will aid in understanding specific disease targets to slow down neurodegeneration. For example, downregulation of key SDCs and GPCs driving amyloid fibrillisation may reduce the build-up of amyloid plaques, thus slowing down disease progression; however, further research is essential to validate this. Recent rodent studies revealed that reducing HS in these brain models improved Aβ clearance and reduced fibril formation (Jen et al. 2009; Poon et al. 2020); however, little has been uncovered regarding HSPG-Aβ interactions in the human brain. This suggests that elucidating the role of HSPGs in HSPG-Aβ interactions may improve our understanding of factors driving neurodegeneration via Aβ accumulation due to impaired clearance. This in turn would enhance the molecular profiles of AD and TBI and provide the foundation for more detailed exploration of HS and HSPGs as molecular targets for use in stem cell and other treatment options.

## Tau and Neurofibrillary Tangles (NFTs)

**Fig. 2:**
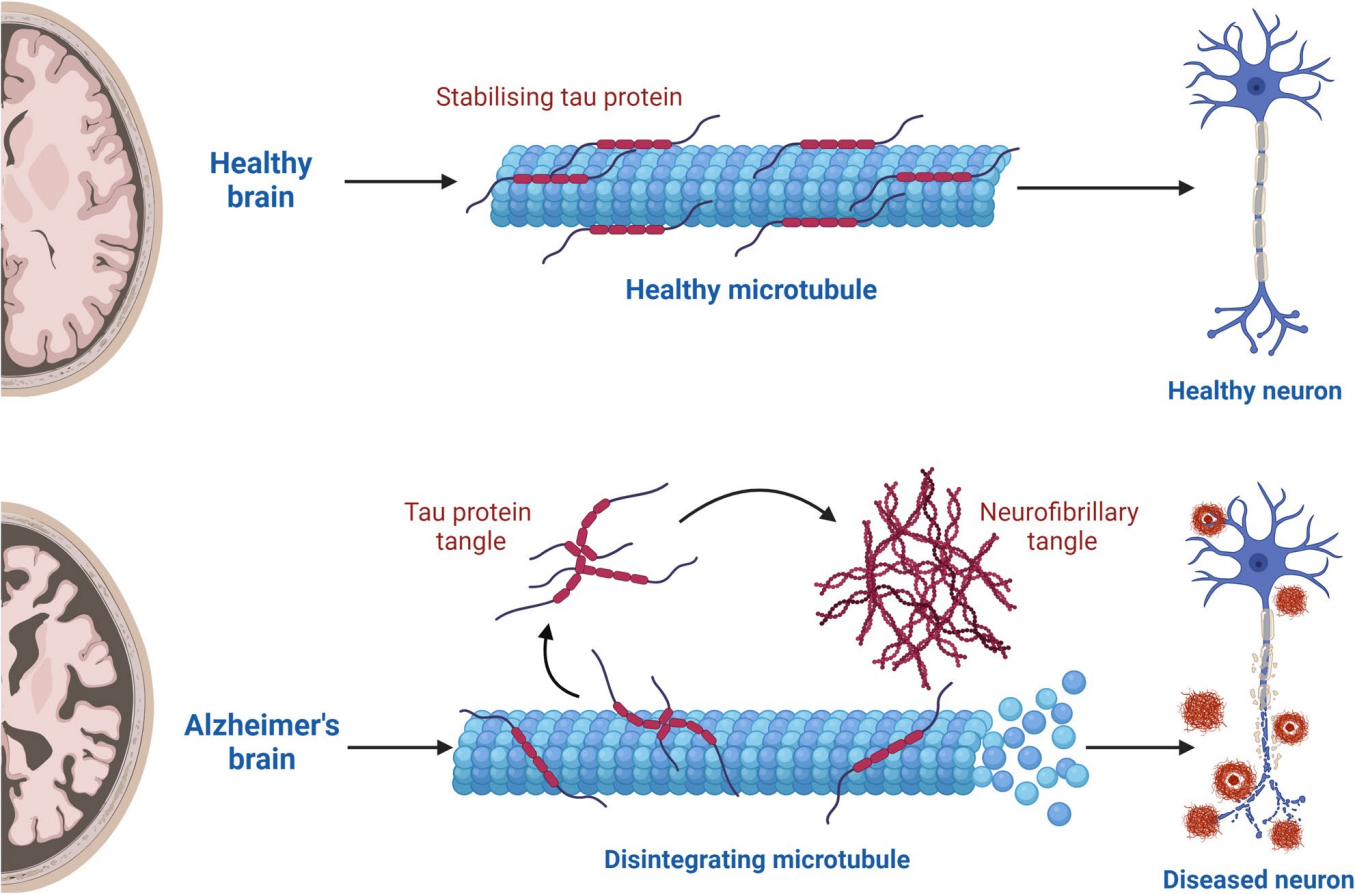
The role of Tau protein in a healthy brain and its pathological accumulation in Alzheimer’s disease (AD). In a healthy brain, Tau protein serves a critical function in maintaining the stability of microtubules, which are abundant in neurons and present in both axons and dendrites. In an AD brain, hyperphosphorylated Tau protein accumulates and forms neurofibrillary tangles (NFTs). The exact mechanism of NFT formation is unknown, but NFTs cause disintegration in microtubules resulting in a diseased neuronal state

In a healthy brain, Tau contributes to microtubular stability and regulation of intracellular trafficking (Congdon and Sigurdsson 2018). Tau pathology in AD begins in the entorhinal cortex and spreads to the hippocampus and other regions of the brain (Lane et al. 2018; Serrano-Pozo et al. 2011). Typically, Tau pathology spares the primary areas controlling sensory, motor and visual functions (DeTure and Dickson 2019; Lane et al. 2018). p-Tau then form the neuropathological feature of AD—aggregating NFTs (Fig. 2) (Metaxas and Kempf 2016).

How NFTs are formed is yet to be determined; however, both intracellular and extracellular NFTs have been found to contain HSPGs (Siedlak et al. 1991). The accumulation of p-Tau is a hallmark of the AD hippocampal-sparing subtype, with this accumulation also observed in other subtypes of AD, including typical AD and limbic-predominant AD (Ferreira et al. 2020; Mohanty et al. 2022; Risacher et al. 2017). Improving our understanding of the Aβ and Tau proteins and their interaction with HSPGs in their accumulation into the plaques associated with AD and TBI could improve diagnostics and treatments for these patients.

## SDCs and GPCs in Alzheimer’s Disease

SDCs and GPCs have been implicated in AD and TBI pathogenesis. As discussed above, the SDC1-3 and GPC1 core proteins detected in amyloid plaques characteristic of AD pathogenesis (van Horssen et al. 2002a, b; Verbeek et al. 1999). Additionally, *SDC3*, which is highly expressed in neuronal cells, has been shown to increase Aβ aggregation in double mutant transgenic mouse models with both an *APP* and *MAPT* mutation (APPSwe-Tau) (Hudák et al. 2022). Elevated *SDC3* expression was also recently reported in neurons and glia of AD patients, suggesting that some HSPGs may be produced in response to Aβ accumulation (Lorente-Gea et al. 2020). The effects of *SDC3* have also been studied in rodent models, with SDC3 deficiency resulting in memory and learning difficulties, symptoms known in both AD and TBI (Lambaerts et al. 2009). In the CNS and during axon guidance, SDC3 has been found to facilitate extracellular Aβ attachment and Aβ fibrillation in SH-SY5Y neuroblastoma cell models (Condomitti and de Wit 2018; Letoha et al. 2019). In addition, increased *SDC3* and *SDC4* expression has been found in post-mortem human AD brains (Grothe et al. 2018; Hudák et al. 2021) and SDC2 and GPC1 to act as Aβ receptors (Jarosz-Griffiths et al. 2016; Letoha et al. 2019; Reinhard et al. 2013). HS-Aβ interactions at these receptor sites contribute to various stages of pathogenesis in AD, including the production, clearance, accumulation, aggregation and toxicity of Aβ (Cui et al. 2013; Liu et al. 2016; Reinhard et al. 2013). Recently, it was suggested that *SDC1* expression may provide an indication of the severity of TBI, with higher expression correlating with poorer outcomes in severely injured patients (Gonzalez Rodriguez et al. 2018; Xie et al. 2024). Impaired neurogenesis is one of the first pathological signs of AD, making the SDC HSPG core proteins important subjects of current AD research (Disouky and Lazarov 2021; Zhang et al. 2014).

The core protein and the HS chains decorating GPC1 have been found to be crucial for brain function, with reduced *GPC1* gene expression resulting in decreased brain size by up to 30% along with increased Aβ accumulation in mouse models (Jen et al. 2009; Ohmi et al. 2011). Membrane-bound interactions between the GPC1 and Aβ proteins have also been documented in vivo*,* with Aβ bound by GPC1 potentially facilitated by HS GAGs (Cheng et al. 2013; Reinhard et al. 2013). Additionally, while no direct evidence demonstrates a specific role of GPCs in TBI, a cryoinjury mouse model of TBI has suggested increased *GPC1* expression following TBI in the injured mouse brain tissue by in situ hybridisation (Hagino et al. 2003). Recently, the regulatory roles of GPC1 and GPC4 have been confirmed in human neural differentiation (Oikari et al. 2020). The expression of these HSPGs is necessary for neuronal differentiation in human neural stem cells (NSCs) and was found to have altered expression when cultures were augmented with brain-derived neurotrophic factor (BDNF) and platelet-derived growth factor (PDGF), suggesting that GPCs may be novel neuronal lineage markers (Oikari et al. 2020). Elucidating the specific roles of the SDC and GPC core proteins in AD pathogenesis will likely help identify how these ubiquitous proteins within the extracellular microenvironment may be targeted, for example, in conjunction with amyloid clearing therapies.

## Other HSPGs and HS Related Genes in Alzheimer’s Disease

The focus of this review is primarily the SDCs and GPCs, however, to more broadly understand HSPG involvement in AD, it is essential to examine how secreted HSPGs may also contribute to AD pathogenesis. As an example, perlecan has been shown to accumulate in the fibrillar Aβ found in human AD patient brains (Perl 2010; van Horssen et al. 2003). The binding of perlecan to predominant isoforms of Aβ may accelerate Aβ fibril formation to stabilise the fibrils, contributing to AD pathogenesis (van Horssen et al. 2003; Lavorgna et al. 2023). In addition, earlier studies indicated that the secreted HSPG agrin may enable organisation of amyloid plaques as well as increase the immunoreactivity observed in AD (Berzin et al. 2000). Type XVIII collagen has also been associated with amyloid deposits and plaques characteristic of AD (Salza et al. 2015; van Horssen et al. 2002a, b). As such, secreted HSPGs likely pose an interesting avenue of research into neurodegenerative diseases such as AD.

HS biosynthesis enzymes have also been identified as contributors to AD pathogenesis. Recent mouse models have identified 3-*O*-sulfotransferase isoform 1 (3-OST-1), encoded by the Heparan Sulfate-Glucosamine 3-Sulfotransferase 1 (*HS3ST1*) gene, in Tau cellular uptake via generation of specific 3-*O*-sulfation sites on HS chains (Wang et al. 2023). Wang et al. (2023) suggest overexpression of the *HS3ST1* gene, a member of the HS biosynthetic enzyme family, may enhance Tau pathology spread, presenting a novel therapeutic target for AD (Wang et al. 2023). More recently, several genes involved in HS biosynthesis, including *EXT1* and Sulfatase 2 (*SULF2*), were found to be downregulated in AD mouse models. In contrast, in this same model, several late-stage HS biosynthesis genes, including *HS3STs* and Sulfatase 1 (*SULF1*) were shown to be upregulated (McMillan et al. 2023). The extracellular sulfatases SULF1 and SULF2, encoded by *SULF1* and *SULF2,* respectively, were also investigated with findings suggesting the HS moieties binding to Aβ contained 6-*O*-sulfation, further highlighting the involvement of HSPGs and HS biosynthesis in AD (McMillan et al. 2023).

In 1989 it was proposed by Snow and Wight that HSPGs were central to the initiation of AD pathogenesis, with subsequent research further validating this initial hypothesis (Snow et al. 2021). In 2021, Snow et al. proposed a new HSPG-AD hypothesis, where increased HSPGs, GAGs and HS sulfation as well as HS degradation in neural cells occur due to a myriad of factors that all contribute to AD pathogenesis. Similarly, the hypothesis posits that increased HS levels accelerate neurodegeneration in AD via increased Aβ and Tau protein aggregation, as well as Aβ plaque formation and hinderance of Aβ fibril and Tau tangle clearance (Liu et al. 2016; Snow et al. 2021). Extensive research into this hypothesis continues, however, the specific interactions between HSPGs and key stages of AD pathogenesis are yet to be fully elucidated.

As such, with HSPGs and HS biosynthesis genes and proteins implicated in the pathogenesis of AD, it is likely that SDCs and GPCS are not the only contributors to AD, although they are the primary focus of this review. Understanding the interactions between HSPG-related genes, including the SDCs and GPCs, Aβ accumulation, Tau pathology and other features of neural lineage specification may facilitate the development of a molecular profile of AD and TBI to identify key signalling events of early diagnosis along with more targeted therapies.

## Alzheimer’s Disease Risk Genes

AD is typically classified into two subtypes, familial AD (FAD) and sporadic AD (SAD), where FAD is most often early onset (< 65 years) and SAD is late onset (> 65 years) (Lista et al. 2015). Mutations in the *APP*, presenilin-1 (*PSEN1*) and presenilin-2 (*PSEN2*) genes have been identified to be causative in AD, particularly FAD, while apolipoprotein E (*APOE*) and microtubule-associated protein Tau (*MAPT)* genes are thought to increase the risk of late onset AD (Lanoiselee et al. 2017). These AD risk genes and their products each have specific connections to HS and HSPGs, as briefly discussed below.

The second most common AD-related gene, the *APP* gene, located on chromosome 21*,* encodes for APP, a member of a protein family including the amyloid precursor-like proteins (APLP1 and APLP2) (Lanoiselee et al. 2017; O’Brien and Wong 2011). APP overexpression improves cellular health and growth (O’Brien and Wong 2011) with alternate splicing of *APP* thought to escalate Aβ accumulation and increase the risk of AD development (O’Brien and Wong 2011; Xiao et al. 2021). Soluble APP (sAPP) interacts with cell surface HSPGs, namely SDC2 and GPC1, with overexpression of these HSPG core proteins shown to have a higher affinity for the sAPP receptor in rodent models (Reinhard et al. 2013). The specific interactions between APP and HSPGs need to be further studied to fully understand the extent of SDC and GPC involvement as well as the HS GAG chains affiliated with these proteins.

The *APOE* gene, located on chromosome 19, encodes for Apolipoprotein E (ApoE) and is currently classified as the biggest risk factor in SAD development (Lane et al. 2018; Liu et al. 2013). Within the CNS, ApoE has the vital function of lipid distribution (Frieden et al. 2017). The most studied *APOE* variants refer to the variation of amino acids at positions 112 and 158 (Frieden et al. 2017; Liu et al. 2013; Marais 2019) and include ApoE2 (protective), ApoE3 (common allele) and ApoE4 (risk). The ApoE2 variant has been found to be beneficial for neurological and cardiovascular health, while the ApoE4 variant is associated with higher risk of AD development (Liu et al. 2013; Marais 2019). Additionally, several studies have also shown a link between the presence of the ApoE4 allele and poorer outcomes for TBI patients (Diaz-Arrastia et al. 2003; McFadyen et al. 2021; Teasdale et al. 1997). Interactions between ApoE and HSPGs, specifically *SDC3*, have been linked to increased Aβ uptake and aggregation (Hudák et al. 2021). Additionally, overexpression of *GPC4* was found in ApoE4 carrier patient post-mortem brain, with ApoE4 surface trafficking via GPC4 gateways suggested to increase Tauopathy (Saroja et al. 2022).

The *MAPT* gene, located on chromosome 17, encodes the Tau protein, which, when hyperphosphorylated, leads to fibrillation and aggregation of NFTs (Caillet-Boudin et al. 2015; Strang et al. 2019). Up to 50 mutations in the *MAPT* gene have to date been demonstrated to increase the risk of select Tauopathies, a term used to describe a group of neurodegenerative disorders characterised by Tau protein accumulation in the brain, which includes AD (Strang et al. 2019). Presence of 3-*O*- and 6-*O*-sulfation have also been implicated in HS-mediated Tau protein internalisation and phosphorylation (Rauch et al. 2018; Sepulveda-Diaz et al. 2015). While in vitro evidence suggests HS and its sulfation patterns contribute to Tau aggregation, the extent of HSPG interactions in vivo remains unclear (Mah et al. 2021).

Mutations in the *PSEN1* gene, located on chromosome 14, encodes the presenilin-1 (PS1) protein, and have been found to be the main causative mutations in FAD with *PSEN1* considered the most common AD-related gene in this disorder (Kelleher and Shen 2017; Lanoiselee et al. 2017). *PSEN2*, located on chromosome 1 (Kumar-Singh et al. 2006), encodes the presenilin-2 (PS2) protein (Kabir et al. 2020; Lanoiselee et al. 2017). Both PS1 and PS2 catalyse the cleavage of APP and production of Aβ (Lanoiselee et al. 2017; Xiao et al. 2021). Mutations in these genes also have the potential to initiate greater production of toxic Aβ42 fragments, contributing to AD pathogenesis (Kabir et al. 2020; Kelleher and Shen 2017). Interestingly, *SDC3* is a known substrate for the PS/γ-secretase complex (Schulz et al. 2003), however, little is known about the interactions between the PSENs and HSPGs.

Similar to the PSENs, few interactions between HSPGs and A disintegrin and metalloprotease10 (ADAM10) encoded by the similarly named *ADAM10* gene, have been identified. ADAM10 is a key enzyme in the non-amyloidogenic pathway of APP processing and may be another AD therapeutic target (Khezri et al. 2023). Elevated BACE1 activity and decreased *ADAM10* expression in late onset AD, is thought to contribute to increased Aβ production and neurodegeneration (Suh et al. 2013). Understanding the interactions between these high-risk genes and any connections with HSPGs, identified as contributors to AD risk such as *SDC3* and *GPC4*, will improve our knowledge of drivers in AD and other neurodegenerative diseases.

## Traumatic Brain Injury Increases Risk of Alzheimer’s Disease

TBI is a complex injury caused by major head trauma, such as gunshot wound and motor vehicle accidents (Dams-O’Connor et al. 2016; Ramos-Cejudo et al. 2018). TBI is characterised by vascular dysfunction and hypoxia with consequent ischemia and individuals with brain trauma are likely to experience delayed cognitive decline years after injury (Ramos-Cejudo et al. 2018). The extent of cellular damage and repair processes following a TBI remain elusive due to the diverse array of type and location of TBIs. However, the injury is known to trigger Aβ-genesis and plaque accumulation, and subsequent neurodegeneration may exhibit similar characteristics to those of AD (Abu Hamdeh et al. 2018; O’Callaghan et al. 2008; Veitch et al. 2013).

## Amyloid Beta and Tau

Diffuse axonal injury (DAI) accounts for approximately 70% of TBI cases (Ng and Lee 2019). Patients with DAI have elevated Aβ levels compared to non-DAI injuries (Marklund et al. 2009). Aβ plaques similar to those found in early-stage AD patient brains have been found in approximately 30% of patients of all ages who die suddenly due to TBI (Johnson et al. 2010b). Interestingly, these plaques have also been found in surviving TBI patients (DeKosky et al. 2007). Aβ deposits similar to those seen in AD may occur within hours of TBI with dense plaque formation reported in rare cases (Ikonomovic et al. 2004; Johnson et al. 2010a; Smith et al. 2013). Similar to Aβ plaque formation in TBI, Tau aggregation occurs within days or even hours after the injury is sustained (Edwards et al. 2020). A demonstrated link between Tau aggregates and TBI was found with one third of surviving moderate to severe TBI patients identified to have post-mortem NFT aggregates (Johnson et al. 2012). Mouse models have been used to demonstrate AD-like tauopathies in TBI-induced conditions, supporting a clear link between AD and TBI (Edwards et al. 2020). Mouse models continue to be used as a basis for human models of Aβ and Tau features in AD and TBI. However, utilising human stem cells has the potential to more accurately model the events leading to these neurodegenerative diseases.

## Current Diagnosis and Treatment of AD and TBI

There are currently no effective early diagnostic or treatment options for AD, with diagnosis only occurring once symptoms are established (Tarawneh and Holtzman 2012; Teipel et al. 2022). Current definitive AD diagnosis requires post-mortem brain tissue biopsy and evaluation (Weller and Budson 2018). Diagnosis for living patients is slowly developing and improving in accuracy, via the combination of CSF and positron emission tomography (PET) biomarkers in conjunction with clinical criteria including cognitive function and memory decline (Teipel et al. 2022; Weller and Budson 2018). Currently, CSF can be tested for Aβ42, total Tau and p-Tau protein with up to 90% diagnostic accuracy (Tariciotti et al. 2018; Weller and Budson 2018).

Meanwhile, TBI is assessed and diagnosed with relative accuracy using neurological medical assessment tools, such as the Glasgow Coma Scale (GCS), as well as imaging tools such as computerised tomography (CT) and magnetic resonance imaging (MRI) (Galgano et al. 2017). The severity of injury is challenging to determine and the long-term implications even harder to predict with difficulty in simultaneously targeting multiple aspects of the disease with a single therapeutic approach (Herrmann et al. 2022; Lyu et al. 2022). The development of diagnostic techniques via cellular and genomic research to investigate early symptoms, risk factors or underlying pathology of both AD and TBI may provide opportunities for earlier and more targeted treatment options. Insight into the initiation events of neurodegeneration at the earliest stages, will provide avenues for direct intervention, potentially preventing or slowing down disease progression. This in turn may contribute to more positive patient outcomes.

## Wnt Signalling in hMSCs

The SDC and GPC HSPGs regulate several cellular signalling pathways, which in turn regulate neural lineage specification (Okolicsanyi et al. 2014b; Ornitz and Itoh 2015). Specifically, the Wnt pathway has documented interactions with HSPGs and influences neural regeneration (Colombres et al. 2008; Oikari et al. 2016). The structure of the HS chain determines its ligand binding activity and other functions of these proteoglycans (Sarrazin et al. 2011). As identified earlier, heparin binding ligands include the Wnt and FGF growth factor families, as well as BDNF and PDGF (Ling et al. 2009).

Wnt signalling pathways are a group of conserved signalling pathways regulating cellular development and in adult stem cells, including proliferation, apoptosis, migration and polarity (Ai et al. 2003; Palomer et al. 2019; Sethi and Vidal-Puig 2010). The Wnt pathways are broadly classified into the canonical and non-canonical pathways (Ackers and Malgor 2018; Liu et al. 2022). Wnt signalling, activated through Wnt and FGF protein interactions with structurally diverse HSPGs, is crucial during neurogenesis and neural development (Ai et al. 2003; Pulsipher et al. 2015). Dysregulation of this signalling has been linked to cerebral synaptic decline in memory loss, dementias and other neurological disorders such as Parkinson’s disease (Berwick and Harvey 2012; Palomer et al. 2019).

The cysteine rich glycol-lipoprotein Wnt proteins function by binding to the Frizzled (FZD) family of receptors and low-density lipoprotein receptor-related protein 5 or 6 (LRP5/6) co-receptors (Boland et al. 2004; Ren et al. 2021). Stabilisation and accumulation of β-catenin occurs followed by its translocation to the nucleus to activate transcription of target genes (Hua et al. 2018; Ren et al. 2021). Canonical Wnt/β-catenin signalling has been suggested to regulate hMSC proliferation with Wnt expression levels influenced by cell density and lineage commitment of hMSCs (Alfaro et al. 2011). Wnt signalling has also been reported to regulate hMSC self-renewal and stem cell lineage differentiation potential (Ai et al. 2003; Ling et al. 2009; Xu et al. 2016). Specifically, Wnt signalling activation inhibits differentiation of hMSCs toward osteogenic and adipogenic lineages, while activation of the pathway has been linked to neuronal differentiation in hMSCs (Liu et al. 2009; Tsai et al. 2014). Interestingly, downregulation of Wnt signalling (Wnt ligands *WNT2B*, *WNT6* and *WNT7a* and FZD receptors *FZD2* and *FZD3)* has been observed in the aging brain (Folke et al. 2019). This may increase synapse vulnerability characteristic of AD pathogenesis, with potential indirect effects influencing Aβ aggregation (Liu et al. 2014; Palomer et al. 2019).

The non-canonical Wnt pathway is activated by Wnt ligands binding to FZD receptors and other co-receptors, such as Ror2 and Ryk (Ackers and Malgor 2018). This leads to the activation of several signalling pathways, including the planar cell polarity pathway, the Wnt/Ca2 + pathway and the Wnt/β-catenin-independent pathway (Komiya and Habas 2008). Non-canonical Wnt signalling mediates developmental processes, including cell migration, axon guidance and tissue patterning (Mehta et al. 2021). Recent work has shown that HS chains decorating HSPG core proteins, including GPC1, GPC3 and GPC5, bind to Wnt, negatively regulating the protein, resulting in inhibition and downregulation of canonical Wnt signalling (Annaval et al. 2020).

Key growth factors, such as BDNF and PDGF, have been identified to influence neurogenesis and interact with HSPGs to subsequently activate signalling pathways including the Wnt pathway (Houlton et al. 2019). BDNF is a member of the neurotrophin family and binds to tropomyosin-related kinase receptors, activating downstream signalling cascades, including the Wnt/β-catenin signalling pathway (Li et al. 2009). Through stimulation of neuronal growth, cellular differentiation and maturation, BDNF is commonly used in neuronal differentiation of human pluripotent stem cells, including induced pluripotent stem cells (iPSCs) (Colucci-D’Amato et al. 2020; Kanato et al. 2009; Tao and Zhang 2016) making BDNF an ideal growth factor for hMSC growth stimulation and differentiation. PDGF binds to members of the receptor tyrosine kinase (RTK) family (Chen et al. 2013; Moore et al. 2014) to regulate cell proliferation, neuronal development and stem cell marker expression (Funa and Sasahara 2014). The PDGF family of growth factors consists of several ligands (PDGF-A, -B, -C and -D) and receptor isoforms (PDGFRα and PDGFRβ), which can act as mitogens for cells of mesenchymal origin (Funa and Sasahara 2014). In NSC models, PDGF-A has been identified to induce differentiation to oligodendrocytes (Douvaras and Fossati 2015; Yan et al. 2013) and several studies report PDGF-B to have neurogenic stimulatory effects in the rodent model in vivo and in vitro, similar to effects established for BDNF (Mohapel et al. 2005; Yang et al. 2013; Yao et al. 2012). The reported binding of PDGF-B to HS, as well as its reported neurogenic effects in the rodent, suggest that PDGF-B could provide an additional means to control human neurogenesis. The effects of both BDNF PDGF on human neurogenesis and Wnt signalling activation, as well as their interaction with the SDCs and GPCs, have not yet been fully explored, requiring further investigations. Exploring intrinsic regulators of neurogenesis, including signalling pathways such as Wnt and growth factor interactions (BDNF, PDGF) in hMSCs will aid the development and understanding of the processes to differentiate these cells into functional neural cells.

## hMSCs as Stem Cell Therapy Models

Stem cell therapy and neurogenesis may provide a potential method to replace injured or dead neural cells that are a feature of AD and TBI (Abdelnour et al. 2022; Neirinckx et al. 2013). The role of stem cell therapy would be to aid in the preservation of existing functional neural cells and to potentially restore and reintegrate new cells to areas of neurodegeneration for improved or repaired functionality. Various stem cell models, including hMSCs, the focus of this review, are being researched in context of neural regeneration and the use of stem cell models for their repair. hMSCs are adult-derived stem cells that have the ability to self-renew, proliferate and differentiate into various mesenchymal cell types, including osteoblasts, adipocytes and chondrocytes, but also cells of neural lineages (Ullah et al. 2015). They are commonly isolated from the bone marrow of the iliac crest, but can also be isolated from dental pulp, umbilical cord blood, adipose tissue, trabecular bone and the placenta (DiGirolamo et al. 1999; Ma 2010). hMSC multilineage capacity including differentiation into neurons, astrocytes and oligodendrocytes has been demonstrated during extended culture in vitro (Okolicsanyi et al. 2015; Yu et al. 2017). hMSCs express various markers of stemness, the molecular basis of a stem cell’s ability to self-renew and generate differentiated cells, along with specific neural lineage markers such as Nestin (*NES*), glial fibrillary acidic protein (*GFAP*) and βIII–tubulin (*TUBB3*), indicating hMSC potential in neural differentiation (Okolicsanyi et al. 2015, 2014a). In murine models, MSCs have been demonstrated to migrate to the brain and differentiate into glial-like cells after transplantation (Mathot et al. 2020). Recent literature reports minimal side effects and safety concerns in small scale human clinical trials applying both autologous and allogeneic hMSCs to treat neurological diseases such as TBI and stroke. However, clinical outcomes have only demonstrated marginal improvements (Andrzejewska et al. 2021; Tian et al. 2013). While hMSCs are a potential promising candidate for use in stem cell therapies for neurodegeneration, it is evident that significant research needs to be undertaken to elucidate the mechanisms driving neurogenesis and how these may be employed to treat areas of damage.

## Improving hMSC Neurogenerative Potential: Opportunities and Challenges

Neuroregenerative therapy research is yet to reach its full potential, posing a myriad of challenges for both research and therapeutic applications. The challenges are primarily rooted in our limited understanding of the complex processes behind the neurological damage that occurs in both in AD and TBI (Feng and Gao 2012). Without comprehensive understanding of the factors driving disease, they cannot be used as targets to prevent, slow down or cure localised damage. Additionally, while stem cell therapies and neurogenesis show promising potential in the treatment of AD and TBI, a significant knowledge gap remains. One of the challenges posed is the availability of human cell stem cell models that are applicable for both ex vivo research, as well as clinical use due to complications such as stem cell rejection (Guy and Offen 2020). Further challenges exist surrounding the terminal differentiation of hMSCs into specific neural lineages and the comprehensive characterisation of their functional capabilities. Although hMSCs have been successfully induced to form neural-like progenitor cells known as hMSC-INs (Okolicsanyi et al. 2018), limited research has been conducted on the process of terminally differentiating these cells in sufficient quantities along with functionality tests on the differentiated cells. These gaps, however, provide an opportunity for the development of hMSC-based models to study healthy neural lineage specification in various populations from a diverse range of donors, as well as the development of relevant human cellular models of AD or TBI. Understanding the differentiation potential and functional properties of hMSCs as well as how HSPG, growth factor and signalling pathway interactions influence neural differentiation processes is crucial for evaluating their suitability as a regenerative therapy. Further investigation is required to explore the differentiation process, identify specific markers indicative of neural lineages, and assess the functional capabilities of terminally differentiated hMSCs.

There is also a need to further elucidate how SDCs and GPCs mediate neural lineage specification and their interactions with crucial signalling pathways. The precise mechanisms through which SDCs and GPCs regulate cellular signalling pathways of neural lineage specification, particularly their interactions with the Wnt and FGF pathways, requires further investigation. It is also an imperative to decipher the specific interactions between HS chains and various growth factors in these pathways in neural regeneration and lineage specification. These interactions influence neural regeneration and are critical to the therapeutic potential of hMSCs in more effective treatment strategies. Additionally, investigating the mechanisms by which HSPGs regulate protein aggregation may provide new and novel avenues of diagnosis as well as develop effective therapies for AD, TBI and other neurodegenerative disorders.

## Summary

Stem cell therapy has the potential to revolutionise AD treatment via neurogenesis. HSPGs play a vital role in neurogenesis from healthy stem cells, making them essential targets for AD research (Ravikumar et al. 2020; Yu et al. 2017). Identifying the genetic contributors and associated expression changes of neurodegeneration in AD and TBI is integral to developing effective disease-specific treatments. hMSCs are ideal therapeutic candidates for the treatment of neurodegenerative diseases due to their relative ease of isolation and extensive in vitro expansive potential. HSPGs, a diverse family of macromolecules, are decorated with multiple GAG side chains that enable interactions with various protein families and ligands. HSPGs of interest include the cell membrane bound SDC and the GPI-anchored GPCs due to their known and postulated contributions to cellular regulation. SDCs mediate angiogenesis and embryonic growth, while GPCs mediate signalling activity as well as a myriad of other essential functions. Further elucidation of how these HSPGs control these events in neurogenesis will likely improve the efficacy of hMSC regenerative applications in treating AD and TBI.

## Data Availability

Not applicable.
